# A new paradigm of bidirectional regulation of the gut–spinal cord axis

**DOI:** 10.4103/NRR.NRR-D-25-01016

**Published:** 2025-11-25

**Authors:** Songzhi Ni, Kai Chen, Haojue Wang, Shenyuan Chen, Yuanyu Qiu, Tianjiao Wang, Fengfeng Mo, Shige Wang, Bo Li, Yushu Bai, Jiulong Zhao, Xiao Zhai, Zhaoshen Li

**Affiliations:** 1 Department of Orthopedics, Shanghai Changhai Hospital, Shanghai, China; 2 Zhenjiang Key Laboratory of High Technology Research on sEVs Foundation and Transformation Application, School of Medicine, Jiangsu University, Zhenjiang, Jiangsu Province, China; 3 Department of Gynecology and Obstetrics, the Affiliated Jiangyin Hospital of Southeast University, Jiangyin, Jiangsu Province, China; 4 Department of Gastroenterology, Shanghai Changhai Hospital, Shanghai, China; 5 National Digestive Endoscopy Improvement System, Shanghai, China; 6 Department of Naval Nutrition and Food Hygiene, Naval Medical University, Shanghai, China; 7 Shanghai Key Laboratory of Nautical Medicine and Translation of Drugs and Medical Devices, Shanghai Changhai Hospital, Shanghai, China; 8 School of Materials and Chemistry, University of Shanghai for Science and Technology, Shanghai, China; 9 Department of Orthopedics, Shanghai Changzheng Hospital, Shanghai, China

**Keywords:** autonomic regulation, biomarkers, enteric nervous system, gut microbiota, host-microbe interaction, intestinal barrier, microbial metabolites, multi-omics approaches, neurodegeneration, neuroinflammation

## Abstract

The bidirectional interactions of spinal cord injury, multiple sclerosis, and amyotrophic lateral sclerosis with the gut operate through a distinct gut–spinal cord axis, rather than being fully explained by the conventional gut–brain axis. The spinal cord, with its unique anatomical and physiological features, serves as a central hub of communication. The gut and spinal cord communicate through various pathways, including the immune system and the autonomic and enteric nervous systems. This review summarizes existing clinical and basic research on the relationship between gut homeostasis and spinal cord diseases. First, we present findings from epidemiological studies showing that patients with spinal cord disorders often exhibit altered gut function, which may be influenced by antibiotic exposure and environmental factors. Second, we review the key physiological and anatomical structures of the gut-spinal cord axis, including the intestinal barrier, gut microbiota, and enteric nervous system, all of which are involved in maintaining gut health, as well as sensory neurons, motor neurons, and interneurons in spinal nerve regulation. Third, we describe the roles of the three axes (microbial, immune, and neural) in bidirectional regulation and their pathological mechanisms. Moreover, vicious cycles involving these axes can exacerbate spinal cord disorders. Fourth, we outline potential biomarkers in the gut–spinal cord axis, such as uridine, hypoxanthine, and 5-methoxytryptophan. Fifth, we propose several treatment strategies with potential clinical applications, including fecal microbiota transplantation and the use of probiotics and prebiotics. Finally, this review emphasizes the gut–spinal cord axis as a promising therapeutic target, highlighting the need for multi-omics integration, longitudinal cohort studies, and individualized interventions to resolve existing debates. Overall, the recognition of the gut–spinal cord axis provides a conceptual shift that extends beyond the gut–brain framework.

**Facts**

• The recognition of the gut-spinal cord axis represents a conceptual shift that goes beyond the gut–brain framework.

• Patients with spinal cord disorders often present with altered gut function, which may be influenced by antibiotic exposure and environmental factors.

• The core components of the gut–spinal cord axis include the intestinal barrier, gut microbiota, and enteric nervous system, all of which work together to maintain intestinal homeostasis.

**Open questions**

• How do the microbial, immune, and neural axes specifically interact in the bidirectional regulation of the gut–spinal cord axis, and how do the vicious cycles among these axes exacerbate the pathological progression of spinal cord diseases?

• How can potential biomarkers of the gut–spinal cord axis be validated through multi-omics integration to determine their clinical applicability in the diagnosis, prognosis assessment, and treatment outcomes of spinal cord diseases?

• How can individualized intervention strategies, such as fecal microbiota transplantation and the use of probiotics and prebiotics, be optimized in the treatment of spinal cord diseases to address existing controversies in gut–spinal cord axis research and improve patients’ quality of life and clinical outcomes?

## Introduction

**Figure 1:**
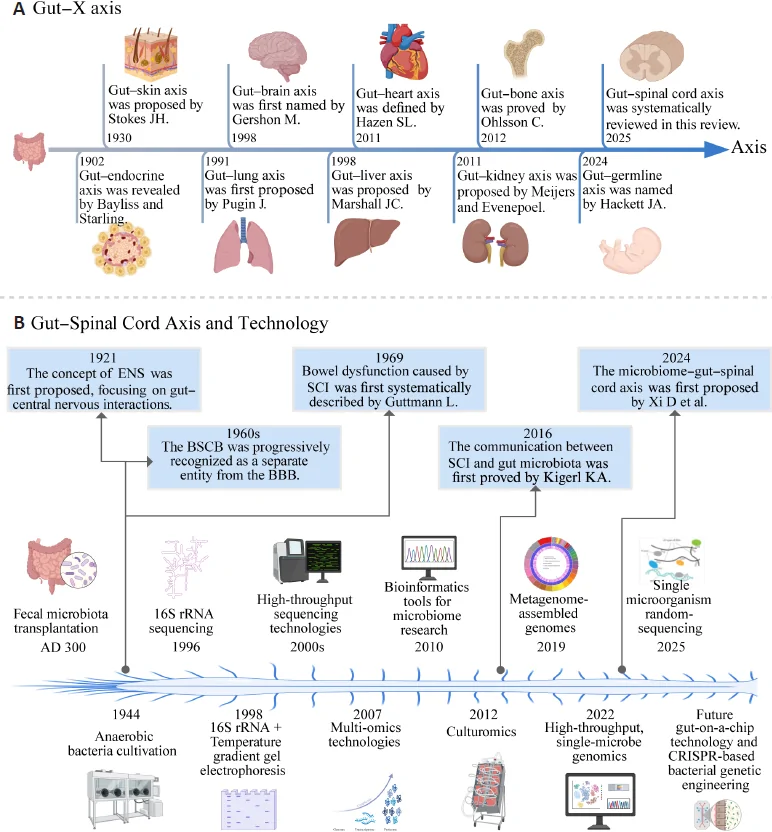
Timeline of the milestone events. (A) Timeline of the milestone events of gut–X axis. In 1902, Bayliss and Starling identified the “gut–endocrine axis.” In 1930, Stokes JH introduced the “gut–skin axis” (Stokes, 1930). In 1991, Pugin et al. coined the “gut–lung axis,” Gershon (1998) formally proposed the “gut–brain axis” in his work *The Second Brain* in 1998, and in the same year, Marshall (1998) introduced the “gut–liver axis.” Hazen SL pioneered the “gut–heart axis” in 2011 (Wang et al., 2011), while Meijers and Evenepoel (2011) proposed the “gut–kidney axis” that same year. In 2012, Sjögren et al. demonstrated that gut microbiota regulates bone mass. Hackett JA named the “gut–germline axis” in 2024 (Argaw-Denboba et al., 2024). In 2025, this paper reviewed the bidirectional communication between the gut and spinal cord. (B) Timeline of the milestone events of gut–spinal cord axis and technology. The concept of the enteric nervous system (ENS) was introduced in 1921 (Langley, 1921), focusing on gut–brain interactions. In the 1960s, the blood–spinal cord barrier (BSCB) was identified separately from the blood–brain barrier (Brightman and Reese, 1969). In 1969, Ludwig Guttmann described bowel dysfunction in spinal cord injury (SCI). In 2016, Kigerl et al. showed bidirectional effects between SCI and gut microbiota. In 2024, Xi et al. proposed the “gut microbiota–gut spinal cord axis.” Technological advances include Robert E. Hungate’s roll-tube method for anaerobic bacteria cultivation (Hungate, 1944), Wilson Blitchington’s 16S rRNA sequencing of fecal bacteria (Wilson and Blitchington, 1996), and Willem de Vos’s discovery of microbiota specificity (Zoetendal et al., 1998). The 2000s saw the rise of metagenomics and high-throughput sequencing, while Rob Knight’s QIIME tool boosted research (Caporaso et al., 2010). Didier Raoult’s culturomics identified new microbes (Lagier et al., 2012), and MAGs expanded knowledge on uncultured microbiota (Nayfach et al., 2019). By 2022, single-microbe genomics resolved microbial genomes at strain and single-cell levels. The Single Microorganism Random-sequencing technique will uncover microbial transcriptional heterogeneity (Xu et al., 2025). Future technologies such as gut-on-a-chip and CRISPR-based genetic engineering will enhance gut microbiome research. Created with BioRender.com.

Spinal cord diseases, including spinal cord injury (SCI), multiple sclerosis (MS), and amyotrophic lateral sclerosis (ALS), impose a substantial burden on patients, with few effective therapeutic options available. These conditions often result in irreversible motor and sensory deficits, persistent pain, and a diminished quality of life (Koch-Henriksen and Magyari, 2021). A growing body of evidence indicates that spinal cord function and gut health are mutually regulated and influence each other bidirectionally (Hu et al., 2023; Hamilton et al., 2024; Willits et al., 2024). Recent advances in understanding the regulatory functions of the gut and its microbiota in human diseases have increased awareness of the contribution of the gut to pathophysiological changes in extraintestinal organs. The gut–X axis includes the liver, heart, brain, lungs, kidneys, bones, skin, and the reproductive and endocrine systems (Lin et al., 2025; **Figure 1A**). While previous studies have largely framed the connection between spinal cord disorders and the gut in terms of the gut–brain axis, the spinal cord and gut exhibit fundamentally distinct structures and functions. This review applies the concept of the “gut–spinal cord axis” to better capture the unique interactions between these systems (Kigerl et al., 2020).

In the past century, the gut–spinal cord axis paradigm has advanced substantially (**Figure 1B**). At the beginning of the 20th century, scientists discovered that gastrointestinal disorders often present with neurological symptoms, laying the foundation for understanding the intimate relationship between the gut and the central nervous system (CNS). During this period, researchers began to investigate the complex interactions between the gut and CNS, with particular attention to a key communication pathway that exerts substantial effects on both systems. In 1921, the enteric nervous system (ENS), referred to as the “second brain,” was identified, indicating that the gut can function independently while relaying information to the CNS. This insight has proven crucial in connecting the gut with the spinal cord and establishing the central role of the spinal cord in regulating gut physiology (Besecker et al., 2020) through neuroendocrine, immune-inflammatory, and microbiota-derived metabolic mechanisms (Fan et al., 2022). The role of gut microbiota as a regulator of spinal cord function was also examined, highlighting the great implications of dysbiosis for neural function.

In recent decades, advancements in technology and a greater emphasis on the interactions between the spinal cord and gut have propelled considerable progress in this field. Gastrointestinal dysfunction is a common consequence of SCI, reflecting the clinical importance of the gut–spinal cord axis. One study reported that up to 11% of patients with SCI are readmitted due to gastrointestinal dysfunction, which severely affects their quality of life (O’Connor et al., 2018). This finding underscores the important role of gut microbiota in SCI outcomes and suggests potential therapeutic targets for rehabilitation. Identifying specific microbial metabolites, such as short-chain fatty acids (SCFAs), is particularly significant in patients with SCI (Kigerl et al., 2020). These substances, produced by microbes, interact with the spinal cord and serve as crucial mediators. They are involved in regulating immune responses, maintaining the integrity of the intestinal barrier and blood–spinal cord barrier (BSCB), and influencing inflammatory signaling pathways (Jing et al., 2021). Simultaneously, the rapidly advancing field of gut microbiota research is expected to continue expanding with the assistance of mathematical models. Multi-omics methods, such as metagenomic sequencing and targeted metabolomics, have enhanced our understanding of the gut microbiota in the gut-spinal cord axis and have shown great potential in creating new treatment strategies (Kigerl et al., 2016). Thus, a thorough understanding of the current state and emerging trends of the gut–spinal cord axis is essential for advancing research and clinical strategies for spinal cord disease rehabilitation.

This paper reviews the gut–spinal cord axis and discusses its theory, mechanisms, diagnostics, and treatment strategies. As an emerging field, it indicates the close connection between the gut and the spinal cord and highlights their relevance to health and disease.

## Search Strategy

We initially acquired related keywords and supplemented them with MeSH subject headings from the National Center for Biotechnology Information. Subsequently, we conducted a comprehensive bibliographic search online through the Web of Science Core Collection, using the following search format: TS=((Spinal Cord Injur* OR Multiple Sclerosis OR Amyotrophic Lateral Sclerosis)) AND TS=(Gut OR Intestin* OR Gastrointestin* OR Gastric OR Gastroentero* OR Anal Canal OR Cecum OR Colon OR Colonic OR Rectum OR Ileum OR Duodenum OR Jejunum OR Bowel OR Digestive System) (Jing et al., 2021; Sun et al., 2024). This search was conducted April 26, 2025. The studies included in this narrative review were limited to articles, and a total of 4705 articles were retrieved, including 1847, 2663, and 354 articles related to SCI, MS, and ALS, respectively. Most of the studies cited in this review were published between 2020 and 2025, with the full range spanning 2002–2025.

## Gut–Spinal Cord Axis: Epidemiological Characteristics and Environmental Modulators

### Gut-spinal cord axis and spinal cord injury

SCI, a condition resulting from various pathogenic factors that impair the structure and function of the spinal cord, often leads to dysfunction of the motor, sensory, and autonomic nerves (Deng et al., 2024; Pagan-Rivera et al., 2024; Tang et al., 2025). The molecular and cellular responses at the trauma site after injury create an environment that is detrimental to cell survival and regeneration. These processes, which include necrosis, inflammation, demyelination, axonal transection, apoptosis, and gliosis, collectively hinder the recovery of motor function.

The epidemiology of the gut-spinal cord axis highlights the serious clinical consequences of gut dysfunction following SCI (Hamilton et al., 2024; Willits et al., 2024). Neurogenic bowel dysfunction (NBD) is prevalent among patients with SCI, influencing their physical and psychological health as well as social participation. Its incidence is particularly high in adult male patients with chronic traumatic complete SCI (Zhang et al., 2018). Importantly, NBD is closely associated with gut dysbiosis, which, in the context of SCI, has been linked to considerable reductions in lipid and fatty acid metabolism. Additionally, a study involving 29 patients with SCI found that 21% exhibited small intestinal bacterial overgrowth (Valles et al., 2021). Intestinal dysfunction is a major determinant of health-related quality of life in patients with SCI, with approximately 46.9% of patients experiencing moderate-to-severe NBD. Furthermore, a predictive model based on 1250 patients with traumatic SCI indicated that the International Spinal Cord Injury Classification total motor score predicts independent and reliable bowel management 1 year post-injury (Pavese et al., 2019). This finding provides a critical basis for clinical consultation, rehabilitation planning, and patient stratification.

Gut dysbiosis may play an important role in controlling inflammatory reactions and facilitating neural regeneration following SCI. For instance, in the T8-10 traumatic SCI mouse model, gut dysbiosis after SCI leads to an increase in the abundance of pro-inflammatory bacteria such as Shigella and Bacteroides, along with a decrease in anti-inflammatory bacteria such as Lactobacillus (Kang et al., 2023). SCI alters the composition and functional capacity of gut bacterial and viral communities. In mouse models, SCI has been shown to reduce the relative abundance of beneficial symbiotic bacteria while increasing the abundance of potentially pathogenic species, as well as decreasing the expression of genes involved in tryptophan, vitamin B6, and folate biosynthesis (Du et al., 2021). This dysbiosis is also evident in patients with SCI, who exhibit reduced microbial diversity, fewer beneficial bacteria, and a higher prevalence of harmful bacteria (Li et al., 2022b). It can be driven by SCI-induced reductions in intestinal motility, prolonged transit time, abnormal immune function, and frequent antibiotic use (Cui et al., 2024b).

Moreover, metabolites produced by the disrupted gut microbiota can cross the BSCB into the CNS, triggering neuroinflammation and contributing to secondary damage following SCI. Dysbiosis can compromise the intestinal barrier and elicit neurogenic inflammatory responses, thereby impeding recovery. These mechanisms establish a bidirectional relationship between the gut microbiota and SCI: SCI induces dysbiosis, which, in turn, exacerbates spinal pathology and delays functional recovery. Supporting evidence for this relationship has been obtained from mouse studies, in which depletion of the gut microbiota through antibiotic cocktails mitigated nerve injury–induced mechanical allodynia and hyperalgesia, while recolonization restored pain symptoms (Lee et al., 2023).

### Gut–spinal cord axis and multiple sclerosis

MS is a chronic, immune-mediated, demyelinating disease of the CNS that leads to impaired neural signaling and symptoms such as limb weakness and sensory disturbances. Recent studies have demonstrated that the gut microbiota plays an important role in the pathogenesis of MS (Stoiloudis et al., 2022; Altieri et al., 2023). Patients with MS have unique gut microbial communities. For example, an international study on the microbiome of MS, which involved 576 patients with MS and 1152 genetically unrelated family health controls, found that the proportions of *Acinetobacter*, *Lactobacillus ruttini*, *Hungatella*, and *Escherichia coli* were significantly higher in patients with MS, while those of *Clostridium* and *Bacteroides* decreased (iMSMS Consortium, 2022).

MS is known to present with gut dysbiosis. Notably, most altered bacteria are SCFA-producing species, indicating their potential contribution to the chronic inflammatory characteristics of this disorder (Ordonez-Rodriguez et al., 2023). Thus, future studies should prioritize the characterization and manipulation of MS-associated microbiota for diagnostic and therapeutic applications.

Further analysis of the gut microbiota in MS also revealed specific bacterial ratios associated with this disease. Fecal studies have indicated that *Prevotella copri* predominates in healthy individuals, whereas *Bacteroides* species are more abundant in patients with MS. In experimental autoimmune encephalomyelitis (EAE) models, a reduced ratio of *Bifidobacterium* to *Akkermansia muciniphila* has been shown to be characteristic of the disease, a finding that has been confirmed in patients with MS. This suggests that the *Bifidobacterium* to *Akkermansia muciniphila* ratio may serve as a potential intestinal microbial biomarker for MS (Ghimire et al., 2025).

Given the potential role of the gut microbiota in the development of MS, interventions targeting the gut have gained increasing attention. Clinical studies have explored the effects of approaches including probiotics and fecal microbiota transplantation (FMT). In a randomized, double-blind, placebo-controlled trial involving 60 patients with MS, participants received either probiotic capsules or a placebo for 12 weeks. In comparison with the placebo group, the probiotic group showed better Expanded Disability Status Scale scores and lower anxiety and depression. Additionally, the probiotic group exhibited significant improvements in insulin resistance, the levels of inflammatory markers, high-density lipoprotein, and malondialdehyde, as well as the total cholesterol/high-density lipoprotein ratio (Rahimlou et al., 2022).

### Gut–spinal cord axis and amyotrophic lateral sclerosis

ALS is a chronic and progressive neurological disorder involving both upper and lower motor neurons. Its pathological hallmark is degeneration and death of motor neurons, which progressively deprive patients of muscle control. In recent years, increasing evidence has highlighted the involvement of the gut microbiota in the pathogenesis of ALS. Some studies examining the gut microbiota in patients with ALS have shown that the gut microbiota in these patients differs considerably from that in healthy controls (Zhai et al., 2019; Zeng et al., 2020). In a study that analyzed stool samples from 20 patients with ALS and 20 healthy controls using 16S rRNA sequencing, considerable alterations were observed in the gut microbial composition of patients with ALS. At the phylum level, the findings showed an increase in the abundance of *Bacteroidetes* and some genera, whereas at the genus level, the abundance of *Megamonas* decreased (Zeng et al., 2020). A comparative study of eight patients with ALS and eight healthy controls revealed that healthy individuals exhibited greater diversity and a more even distribution of bacterial and archaeal communities than patients with ALS. In patients with ALS, the proportion of *Firmicutes*/*Bacteroidetes* and *Methanobrevibacter* tended to be higher, while the relative abundance of beneficial microorganisms such as *Faecalibacterium* and *Bacteroides* decreased remarkably (Zhai et al., 2019).

Furthermore, studies on the gut microbiota of patients with ALS have revealed variations among different ALS subtypes. For example, in patients with ALS whose symptoms emerged within 6–15 months, *Fusobacterium* and *Acidobacteria* were found to be more abundant compared to healthy individuals. Additionally, in patients with ALS who had spinal cord problems, increases in the abundance of *Fusobacterium* and *Bacteroides* were more pronounced. Research on alterations in gut microbiota remains at a descriptive level, and as a result, detailed studies on the mechanistic links between changes in the gut microbiota and ALS have not yet been conducted.

The metabolites of intestinal microorganisms may play a considerable role in the pathogenesis of ALS. Investigations into the metabolites of intestinal microorganisms in patients with ALS aim to reveal their relationships with the disease. Studies of fecal metabolites in patients with ALS have indicated a potential decline in digestive and metabolic functions (Hertzberg et al., 2022; Fontdevila et al., 2024). For instance, a comparative study involving eight patients with ALS and eight healthy individuals found that the average concentrations of human endotoxins, SCFAs, NO2-N/NO3-N, and γ-aminobutyric acid in the feces of patients with ALS were 64.2 *vs*. 65.3 EU/mL, 57.5 *vs*. 55.3 μg/mL, 5.7 *vs*. 5.3 ng/mL, and 6.1 *vs*. 5.4 μmol/L, respectively (Zhai et al., 2019). Furthermore, research examining the relationship between the gut microbiota and metabolites in patients with ALS has demonstrated that microbial alterations are closely associated with metabolic changes. Specific bacterial genera correlate with distinct metabolic pathways. In patients with ALS, shifts in certain genera are linked to perturbations in carbon and butyrate metabolism. These findings suggest that the gut microbiota may contribute to ALS pathogenesis by modulating host metabolic pathways (Hertzberg et al., 2022). Studies of the plasma metabolome of patients with ALS have revealed that lipid metabolism-related metabolites may play a significant role in disease progression (Guo et al., 2024, 2025). A longitudinal metabolomics study has indicated that metabolites associated with fatty acids, sphingomyelins, carnitine, and short-chain acylcarnitines correlate with ALS severity and progression (Guo et al., 2025). Network analysis has linked key bacterial amplicon sequence variants to lipid metabolites, and Mendelian randomization has implicated fatty acid/acylcarnitine-related lipids in ALS causality (Guo et al., 2024). Reducing the bacterial load using broad-spectrum antibiotics or transplanting protective bacteria can improve the inflammatory phenotype and survival of mice (Burberry et al., 2020).

Considering the potential role of the gut microbiota in the pathogenesis of ALS, the search for therapeutic targets against intestinal microorganisms has become a key research direction. Studies have shown that regulating the gut microbiota and its metabolites may provide new approaches for the treatment of ALS (Zhang et al., 2017; Gotkine et al., 2020). Certain bacteria are considered potential therapeutic targets. *Akkermansia muciniphila* has been shown to improve the disease condition in ALS mice (Gotkine et al., 2020; Xu et al., 2023), possibly owing to the production of nicotinamide (Gotkine et al., 2020). Similar functional alterations have been observed in patients with ALS, including reduced nicotinamide levels in both plasma and cerebrospinal fluid. Gut microbiota-targeting interventions such as probiotic supplementation and phage therapy have also been explored. In a study of 50 patients with ALS and 50 healthy controls, specific probiotics modulated gut microbiota composition over 6 months, but did not restore microbiota profiles to those of healthy controls or significantly affect disease progression, as measured by the Revised Amyotrophic Lateral Sclerosis Functional Rating Scale (Di Gioia et al., 2020). Further clarification of the specific link between the gut microbiota and the pathogenesis of ALS is expected to reveal more effective therapeutic targets and provide new strategies for the treatment of ALS.

### Popularity characteristics of the gut-spinal cord axis

The epidemiological characteristics of the gut-spinal cord axis vary among populations. In patients with SCI, changes in the gut microbiota are relatively common and are associated with the factors including the severity of the injury and the time since injury (Bannerman et al., 2022). Studies analyzing patients with SCI have found that the diversity and composition of the gut microbiota undergo significant changes after injury, with these changes differing between the subacute and chronic phases (Rong et al., 2021; Zang et al., 2023). During the subacute phase, changes in the gut microbiota are particularly pronounced, with an increase in harmful bacteria (Bazzocchi et al., 2021; Zhong et al., 2025). During the subacute phase (Gur Arie et al., 2024), the relative abundances of *Bacteroidetes*, *Clostridiales*, *Faecalbacterium*, *Ruminococcus*, *Coprococcus*, *Lachnospira*, *Dorea*, *Prevotella*, *Roseburia*, *Atopobium*, *Bifidobacterium*, *Bacteroides*, and *Blautia* increase, whereas those of *Firmicutes* and *Lactobacillus* decrease (Zhong et al., 2025). Inherent variations have been identified in gut microbiota composition across age (Jang et al., 2024), sex (Snigdha et al., 2022), and host habitat (Zhang et al., 2024a), which may affect spinal cord diseases. Nevertheless, the specific functional consequences of these differences on the gut-spinal cord axis remain unexplored and require further investigation (Calcaterra et al., 2022).

### How do environmental factors influence the gut–spinal cord axis?

Environmental factors (e.g., diet, exercise, stress, and sleep) significantly influence the gut-spinal cord axis by altering the structure and function of the gut microbiota. For example, high-fiber diets increase beneficial gut microbiota and enhance SCFAs production, which support the gut-spinal cord axis. In contrast, diets high in fat and sugar can affect the balance of the gut microbiota, which can increase the risk of spinal cord diseases (Zhao et al., 2019). Beneficial bacteria such as *Akkermansia* are correlated with favorable recovery after SCI and can be encouraged through a dietary regimen high in fiber, prebiotics, and probiotics (Valido et al., 2025). Interventions such as FMT have also been demonstrated to correct dysbiosis and facilitate recovery following SCI by modulating the immune response and inflammation. Physical activity is a clear lifestyle modulator of the gut microbiota and, therefore, of the gut–spinal cord axis (Gur Arie et al., 2024). Routine physical activity has been shown to enhance gut microbiota diversity and promote beneficial microbes, reducing systemic inflammation and protecting spinal cord health (Hu et al., 2024). Appropriate management of stress and sufficient restorative sleep are important factors for ensuring healthy gut microbiota. Prolonged stress and sleep deprivation can trigger imbalances leading to adverse inflammatory responses and impeding spinal cord recovery (Musleh-Vega et al., 2022). Thus, leading a healthy lifestyle with a dieting plan, a regular physical activity program, appropriate stress relief measures, and proper sleep cycles could facilitate better performance of the gut-spinal cord axis, achieve better treatment results in patients with SCI, and improve overall spinal health. A more thorough understanding of the influence of these lifestyle activities on the gut–spinal cord axis may open avenues for novel treatments to complement or modify the available treatments for clinically related spinal cord problems.

Environmental pathogen infections and antibiotic utilization also interfere with the gut microbial balance, increasing the risk of spinal cord diseases. Pathogenic infections can trigger inflammatory responses in the CNS, aggravating neurodegenerative processes. Infections trigger local immune cells in the spinal cord and recruit adjacent immune cells, which may cause neuropathological alterations and increase the probability of developing neurodegenerative diseases (Lotz et al., 2021). In SCI, the gut–spinal cord axis plays a major role in regulating the inflammatory response and nerve recovery (Pagan-Rivera et al., 2024), and this inflammatory response would be destructive if the gut is infected. Antibiotic usage is another major factor that can disrupt the gut microbiota. Antibiotics may lead to a reduction in species variety, changes in metabolic function, and the selection of antibiotic-resistant organisms, which can result in conditions such as antibiotic-related diarrhea and recurrent *Clostridium difficile* infections. Gut dysbiosis induced by broad-spectrum antibiotics and SCI has been shown to impair functional recovery (Rong et al., 2021). Furthermore, in the superoxide dismutase 1 (SOD1) ALS model, low-dose antibiotic treatment was shown to accelerate disease progression, reduce the abundance of *Akkermansia* and butyrate-producing bacteria, and promote a neurodegenerative microglial (MGnD) phenotype. The bacterial flora exerts a protective effect by inhibiting the MGnD phenotype and improving the disease (Cox et al., 2022). Therefore, antibiotic exposure, particularly during early life, can profoundly alter gut microbiota, leading to gastrointestinal, immunological, and neurocognitive disturbances. Such dysbiosis may further impair recovery after SCI by disrupting the gut-spinal cord axis and promoting the production of pro-inflammatory metabolites that compromise cell survival and locomotor function (Pagan-Rivera et al., 2024).

## Basic Theories Explaining the Gut–Spinal Cord Axis

**Figure 2:**
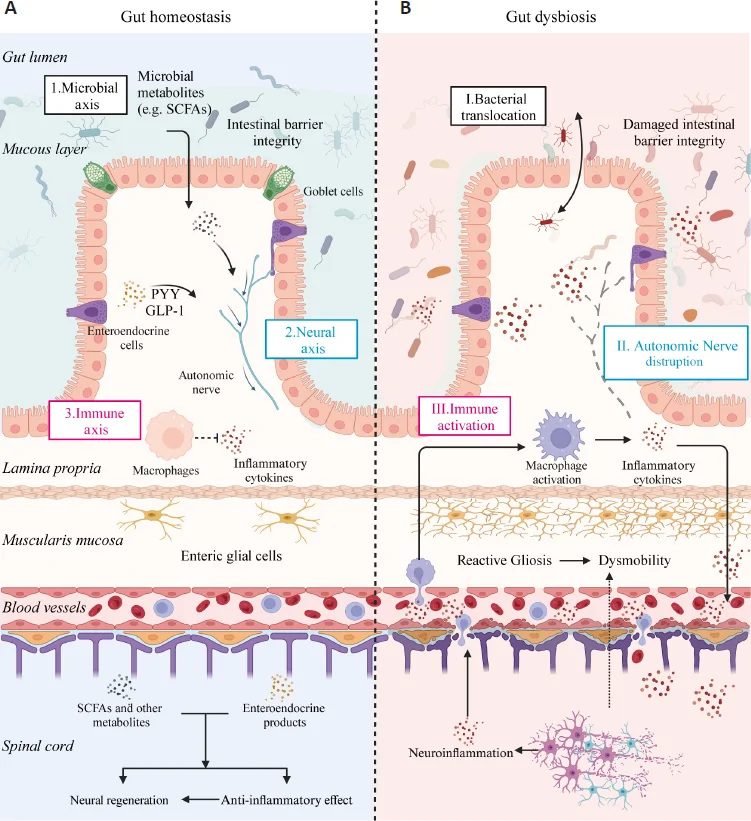
Schematic illustration of the gut-spinal cord axis. (A and B) Three axes in gut–spinal cord interaction in gut homeostasis (A) and gut dysbiosis (B). The microbiota in gut homeostasis interacts with the spinal cord through the transformation of intestinal contents into various metabolites, for instance, SCFAs. SCFAs can cross the intestinal barrier and have a multitude of beneficial impacts on the spinal cord by modifying the metabolism of neurons and microglia. The peripheral nerves innervated by the spinal cord stimulate enteroendocrine cells, which release various hormones to regulate the digestive process. At the same time, the binding of intestinal metabolites to receptors on peripheral nerves indirectly influences the spinal cord. The gastrointestinal tract is the largest immune organ in the human body, and gut homeostasis inhibits the activation of pro-inflammatory cells and the production of inflammatory cytokines. (I) Gut dysbiosis impairs the intestinal barrier and leads to bacterial translocation. This situation then exerts a negative impact on the intestinal parenchyma and spinal cord. (II) Enteroendocrine cell malfunction is caused by autonomic nerve dysfunction and enteric nerve atrophy. (III) Macrophages polarize into a pro-inflammatory state, producing pro-inflammatory cytokines that affect the spinal cord through circulation system. Created with BioRender.com. GLP-1: Glucagon-like peptide-1; PYY: peptide YY; SCFAs: short-chain fatty acids.

The gut-spinal cord axis consists of a bidirectional communication system mediated by specific structures, such as the intestinal barrier, microbiota, and ENS (Kigerl et al., 2016; Pagan-Rivera et al., 2024; **Figure 2**). Gut-derived microbial metabolites (e.g., SCFAs) and immune signals regulate spinal cord function through circulatory or neural pathways, whereas autonomic pathways from the spinal cord control gut motility, secretion, and blood supply. The disruption of these structurally dependent interactions causes gastrointestinal dysfunction (Kigerl et al., 2020).

### Key structures and functions for maintaining gut health

The intestinal barrier consists of mechanical, chemical, immune, and biological mechanisms, all of which play crucial roles in maintaining gut health (Gao et al., 2025). The mechanical barrier relies on intestinal epithelial cells and their tight junctions to prevent pathogens and toxins from invading (Lu et al., 2025). The chemical barrier is maintained by the mucus layer (MUC2 mucin) covering the intestinal lumen, which neutralizes pathogens through antimicrobial peptides (such as defensins) (Chen et al., 2024). In the immune barrier, gut-associated lymphoid tissues (GALT) account for 70% to 90% of human immune cells and can secrete secretory immunoglobulin A to neutralize antigens (Park et al., 2025). The biological barrier includes symbiotic bacteria, which are gut microbial components that engage in mutualistic relationships with the host. These symbiotic bacteria, such as *Bifidobacteria* and *Lactobacilli*, help inhibit pathogenic bacteria through their niche effects by depleting respiratory electron acceptors (such as O_2_ and NO_3_^–^), dietary metabolites, and essential metals, as well as through their metabolic products, such as SCFAs (Pedrosa et al., 2024). Disruption of the intestinal barrier can result in inflammatory bowel disease, leaky gut syndrome, or systemic inflammation, potentially contributing to conditions such as liver diseases and neurodegenerative disorders (Li et al., 2025a; Lista et al., 2025).

The gut microbiota, the largest microbial ecosystem in the human body, consists of over 1000 species of bacteria (approximately 100 trillion), which can be categorized into symbiotic bacteria (> 99%, such as *Bacteroides* and *Bifidobacterium*, which are involved in nutrient metabolism and immune regulation), opportunistic pathogens (such as *Escherichia coli*, which are harmless under normal conditions but potentially pathogenic when imbalanced), and pathogenic bacteria (such as Salmonella, which directly cause infectious diseases) (Tewari and Dey, 2024). An imbalance in the gut microbiota is closely associated with various diseases, including obesity (Carmody and Bisanz, 2023), diabetes (Crudele et al., 2023), depression (Ford et al., 2024), and Parkinson’s disease (Zhang et al., 2023b).

The ENS is often referred to as the “second brain,” and is capable of independently regulating intestinal functions (Hajjeh et al., 2025). It consists of the myenteric and submucosal plexuses. The former controls intestinal peristalsis and muscle contractions, whereas the latter regulates glandular secretion and vascular tone (Dershowitz et al., 2023). The ENS independently controls intestinal motility, including gastric emptying and colonic propulsion, coordinates immune responses through mechanosensors such as Piezo1 (El Baassiri et al., 2024; Loh et al., 2024), preserves intestinal barrier integrity (Sharkey and Mawe, 2023), and communicates bidirectionally with the CNS via the gut–brain axis, affecting emotional and cognitive functions (He et al., 2024).

### Key components and functions of the spinal cord neural network

The spinal cord neural network, a vital component of the CNS, is characterized by its complex structure and diverse functions (Dubuc et al., 2023). Spinal cord neurons include sensory and motor neurons and interneurons, which connect to form complex neural networks (Wu et al., 2024b). The spinal cord neural network primarily regulates sensory and motor functions. Sensory neurons convey information from the body, including touch, pain, and temperature, to the spinal cord, which relays the information to the brain, thereby enabling the perception of environmental changes. Motor neurons receive commands from the brain or intrinsic spinal circuits and control muscle contraction and relaxation to generate movement (Cao et al., 2026). Spinal cord interneurons play a critical role in integrating sensory information and modulating motor output. They process incoming sensory signals and regulate motor neuron activity, ensuring that movements are coordinated and precise (Li et al., 2023).

The gut–spinal cord axis represents a specialized, region-specific regulatory network distinct from the broader gut–brain axis. Its segmental organization allows for compartmentalized control over the gastrointestinal tract, and disruptions in this axis can result in specific gastrointestinal manifestations, such as slowed intestinal peristalsis, constipation, and abdominal distension (Qi et al., 2018). In contrast, brain injury primarily affects the higher neural centers, including the cerebral cortex and brainstem, leading to disturbances in appetite regulation, dysphagia, and delayed gastric emptying. Brain injury can also impair hormonal regulation, reduce the secretion of gastric acid and digestive enzymes, and compromise digestive processes (Hanscom et al., 2021). SCI affects secretory function more indirectly, mainly by altering intestinal peristalsis and thereby affecting the distribution and absorption of digestive juices. The psychological consequences of brain injury, such as anxiety and depression (Goralczyk-Binkowska et al., 2022), can additionally influence gastrointestinal function through neuroendocrine pathways (Person and Keefer, 2021). In comparison, psychological factors have a relatively minor effect on gut function in patients with SCI. Overall, SCI and brain injury differ markedly in their mechanisms, manifestations, and severity of gastrointestinal dysfunction.

## Three Pathological Mechanisms Involving the Gut–Spinal Cord Axis

The gastrointestinal tract and spinal cord are closely interconnected, and disruptions to this relationship can lead to widespread systemic dysfunction. Three interconnected pathological pathways, which are closely related and interact with each other, mainly govern this complex relationship. The first involves the disruption of the normal gut microbiota balance, known as gut dysbiosis, which is characterized by an overgrowth of harmful bacteria and a reduction in beneficial microbes. Another pathway of communication occurs via immune-neural interactions, which help maintain internal homeostasis. Disruption of this crosstalk can lead to chronic inflammation and impair the ability of the body to distinguish harmful pathogens from harmless stimuli. Dysregulation of this system may result in an immune response that targets organs beyond the gut, including the spinal cord. The third pathway involves neural regulation through a complex neural network, especially the autonomic nervous system and ENS. Collectively, these pathways create a vicious cycle that links intestinal dysregulation with spinal cord pathology.

### Gut microbiota dysbiosis in relation to the gut–spinal cord axis

**Figure 3:**
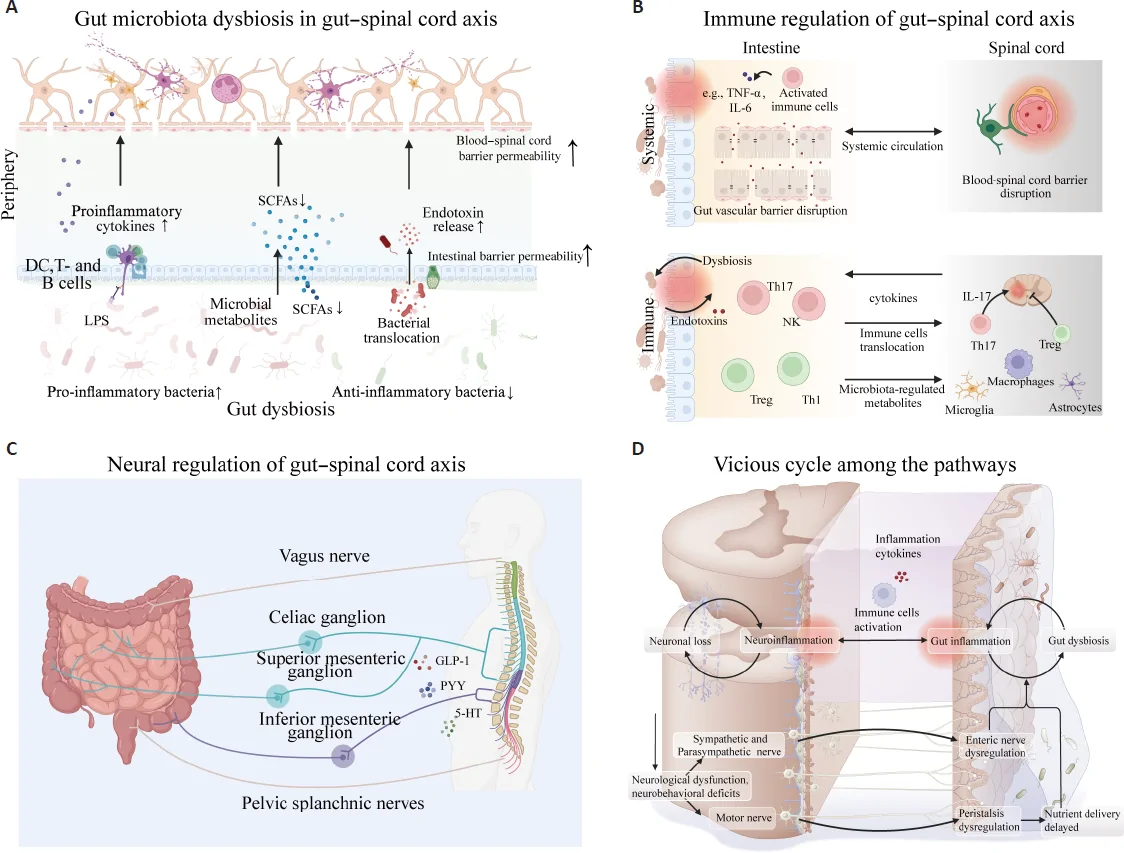
Three pathological mechanisms in the gut–spinal cord axis. (A) Gut microbiota dysbiosis in gut–spinal cord axis is characterized by increased intestinal permeability, bacterial translocation, immune activation in gut-associated lymphoid tissues, and compositional shifts in microbiota. (B) The immune regulatory mechanism of the gut–spinal cord axis is crucial for maintaining homeostasis in both systems. (C) Neural regulation within the gut–spinal cord axis facilitates bidirectional communication between the spinal cord and the gut. (D) Vicious cycle involving the three pathological mechanisms. Created with BioRender.com. 5-HT: 5-Hydroxytryptamine; GLP-1: glucagon-like peptide-1; IL: interleukin; LPS: lipopolysaccharide; PYY: peptide YY; TNF-α: tumor necrosis factor-α.

The gut-spinal cord axis is a critical pathway in microbiota-mediated crosstalk and is characterized by increased intestinal permeability, bacterial translocation, immune activation in GALT, and compositional shifts in the microbiota (Gur Arie et al., 2024; **Figure 3A**). Specifically, SCI elevates the abundance of pro-inflammatory bacteria (e.g., *Shigella*, *Rikenella*, *Staphylococcus*, *Bacteroides*, and *Mucispirillum*) while depleting anti-inflammatory taxa (*Allobaculum*, *Lactobacillus*, and *Sutterella*) (Kang et al., 2023). Notably, an overabundance of *Bacteroides* disrupts the intestinal barrier and exacerbates systemic inflammation (Guo et al., 2021). Chronic SCI further reprograms colonocyte metabolism from β-fatty acid oxidation to anaerobic glycolysis, reducing oxygen consumption and driving microbial community shifts from obligate to facultative anaerobes (Byndloss and Baumler, 2018). This dominance of facultative anaerobes correlates with persistent inflammation in both acute and chronic phases of SCI (Alvarez-Mon et al., 2019).

Altered microbial metabolism in SCI generates aberrant metabolite profiles. Key metabolites such as SCFAs, which are essential for intestinal homeostasis and immunity (Wilson et al., 2023), are depleted due to the antibiotic-induced elimination of SCFA-producing bacteria (e.g., butyrate, propionate, and acetate producers) (Byndloss et al., 2017). This form of metabolite imbalance impairs barrier function and increases permeability and inflammation (Li et al., 2024b). Additionally, microbiota-derived metabolites directly modulate immune responses (Raue et al., 2023), either by exacerbating or mitigating SCI-associated inflammation (Zhu et al., 2024). Emerging evidence indicates that certain metabolites, including indole-3-propionate and melatonin, have neuroprotective properties that potentially facilitate spinal cord recovery (Xie et al., 2023).

#### Phylum Firmicutes

Firmicutes, a major constituent of the human gut microbiota, possess distinctive microbial features, including thick cell walls and a typical Gram-positive staining profile. This phylum has gained increasing attention in SCI studies (Jing et al., 2021, 2023). During the subacute phase of SCI, the relative abundance of Firmicutes decreases, and this trend becomes more pronounced during the chronic phase (Zhong et al., 2025). Furthermore, in patients with ALS, the composition and function of the gut *Firmicutes* differ significantly from those in healthy individuals, and these changes may be linked to disease progression.

The phylum *Firmicutes* plays a bidirectional role in regulating the inflammatory responses in spinal cord diseases. Following SCI, the gut microbiota enters a state of dysbiosis characterized by an increase in pro-inflammatory bacteria such as *Shigella* and *Staphylococcus* and reduced abundance of anti-inflammatory bacteria such as *Lactobacillus*. Studies have shown that certain amino acids, such as l-leucine and l-methionine, produced by *Firmicutes* during metabolism can induce oxidative stress and inflammation, which contribute to secondary damage following SCI (Jing et al., 2021; Liu et al., 2021). For example, certain genera within phylum *Firmicutes* have been shown to activate the NLR family pyrin domain containing 3 (NLRP3) inflammasome. Notably, some *Firmicutes* members, such as *Lactobacillus*, can produce anti-inflammatory compounds under normal conditions. However, when the microbial community is imbalanced, their abundance may fall outside the normal range, potentially diminishing these protective effects (Aube et al., 2014).

#### Genus Bacteroides

The *Bacteroides* genus is a group of Gram-negative, non-spore-forming anaerobic bacteria that are crucial components of the human gut microbiota. These bacteria are widely found on the mucosal surfaces of the gut and mouth. During the pathological progression of SCI, *Bacteroides* exhibits stage-specific changes. A cohort study based on 16S rRNA gene sequencing found that, compared with healthy individuals, the relative abundance of *Bacteroides* in the intestines of patients with SCI significantly increased by approximately 30% during the subacute phase. In the chronic phase, the rate of increase slowed, but the levels remained approximately 20% above the baseline, reflecting the sequential remodeling of the gut microbiota following SCI (Kang et al., 2023).

This change is fundamentally the result of the bidirectional interaction between the spinal cord and intestinal microenvironment. SCI can drive *Bacteroides* dysbiosis through three mechanisms: (1) the systemic stress response after trauma increases intestinal permeability; (2) prolonged bed rest leads to reduced gastrointestinal motility and local ischemia; and (3) the use of broad-spectrum antibiotics directly inhibits bacterial populations. Together, these factors disrupt the physical intestinal barrier (decreased mucus layer thickness) and the chemical environment (pH imbalance and altered nutrient matrix), forcing *Bacteroides* to undergo adaptive fluctuations in population size and species turnover (Kang et al., 2023).

The feedback regulation mechanism of *Bacteroides* in spinal cord pathology is reflected at multiple levels: (1) Immune regulation axis. *Bacteroides* activates the dendritic cells (DCs) in the intestinal lamina propria, promoting the differentiation and expansion of regulatory T cells (Tregs), which, in turn, increases the secretion of anti-inflammatory factors such as transforming growth factor-β and inhibits the cytokine storm of pro-inflammatory factors (such as tumor necrosis factor [TNF]-α and interleukin [IL]-6) in the spinal cord lesion area (Mangalam et al., 2017). (2) Metabolic intervention axis. *Bacteroides* metabolizes dietary fiber to produce SCFAs such as butyrate and propionate. These SCFAs exert concentration-dependent effects: at physiological millimolar levels, they activate the GPR43 receptor to inhibit the nuclear factor (NF)-κB pathway, thereby attenuating inflammatory activation of spinal microglia. However, sudden imbalances in the concentration of SCFAs can cause them to lose their protective effects and even become pro-inflammatory (de Sousa et al., 2025). Additionally, SCFAs can enhance the expression of tight junction proteins in the intestinal epithelium, which reduces endotoxin translocation into the circulatory system and indirectly mitigates oxidative stress damage to spinal neurons (Timofeeva et al., 2025). (3) Regulation of cell migration. Metabolites such as succinic acid from *Bacteroides* can downregulate the expression of L-selectin (CD62L) and integrins in neutrophils, inhibiting their adhesion and infiltration into spinal cord inflammatory lesions and significantly alleviating secondary demyelination (Monteiro et al., 2020). (4) Neural signal modulation. Recent studies suggest that *Bacteroides* metabolites, such as γ-aminobutyric acid (GABA) precursors, may reach the spinal cord through the vagus nerve or blood circulation pathways (Gong et al., 2021; Cao et al., 2022). These substances modulate the plasticity of synaptic transmission by binding to GABA-B receptors on the neuronal surfaces. However, the specific signaling pathways, such as the cyclic adenosine monophosphate (cAMP)-protein kinase A (PKA)-cAMP response element-binding protein (CREB) cascade, remain to be elucidated (Timofeeva et al., 2025).

#### Lactobacillus reuteri

*Lactobacillus reuteri*, which decreases in relative abundance after SCI, has shown the potential to regulate the gut microbiota and promote SCI repair. *Lactobacillus reuteri* can improve neuroinflammatory responses following SCI by regulating tryptophan metabolism. Specifically, this strain promotes the production of indole-3-carbinol, inhibits the M1 polarization of microglia, and reduces the levels of IL-6, IL-1β, and TNF-α in SCI, thereby alleviating neuroinflammation. Additionally, *Lactobacillus reuteri* enhances the expression of the aryl hydrocarbon receptor (AhR) and CYP1A1, activates the AhR signaling pathway, and promotes the synthesis of tight junction proteins, thus repairing intestinal barrier function (Cen et al., 2025). The role of extracellular vesicles (EVs) derived from *Lactobacillus reuteri* in other types of nervous system damage has also been studied. These EVs treat ischemic stroke by clearing excessive reactive oxygen species (ROS), regulating microglia via inhibition of the NF-κB pathway, and reducing neuronal apoptosis (Sun et al., 2025). Thus, *Lactobacillus reuteri* is of great significance in the regulation and amelioration of neuroinflammation following SCI. This strain has the potential to be used as an auxiliary agent for SCI treatment by adjusting the gut microbiota and relevant metabolic pathways. These findings suggest an opportunity for future research to investigate its clinical application potential.

#### Bifidobacterium and acetic acid

*Bifidobacterium*, a probiotic extensively utilized in clinical settings, has recently gained prominence because of its critical role in neurological disorders. During the subacute and chronic phases of SCI, *Bifidobacterium* exhibits increased relative abundance (Zhong et al., 2025). Moreover, *Bifidobacterium* abundance positively correlates with MS progression, whereas a reduced ratio of *Bifidobacterium* to *Akkermansia muciniphila* is associated with greater MS severity (Ghimire et al., 2025; Jiang et al., 2025). Notably, elevated levels of *Bifidobacterium* have also been observed in patients with ALS (Gautam et al., 2025).

The main producers of acetic acid are gut microorganisms, which generate acetic acid through fermentation of dietary fiber. Studies have shown that acetic acid is of great significance in maintaining the intestinal barrier and regulating immune responses (Perez-Reytor et al., 2021; Ma et al., 2024). *Bifidobacterium*, a major probiotic in the gut, generates metabolites such as acetic acid to regulate the balance of the gut microbiota, thus strengthening the function of the intestinal barrier (Li et al., 2024a). Moreover, acetic acid plays a role in neuroprotection by modulating microglial activation; thus, it can offer protection against spinal cord diseases (Zhong et al., 2025).

The metabolic products of *Bifidobacterium* include not only acetic acid but also other useful metabolites. For instance, niacin generated by *Bifidobacterium adolescentis* has been shown to ameliorate skeletal muscle mitochondrial function, thus relieving the symptoms of muscle atrophy (Zhang et al., 2025). Furthermore, indole-3-lactic acid (ILA) generated by *Bifidobacterium infantis* can safeguard intestinal epithelial cells through activation of the AhR and Nrf2 pathways, which can reduce inflammatory responses (Ehrlich et al., 2020).

In summary, *Bifidobacterium* is of great significance in strengthening the function of the intestinal barrier through the production of metabolites such as acetic acid. These findings offer novel insights into the role of the gut microbiota in neurological disorders.

### Immune regulation of the gut–spinal cord axis

Homeostasis of both systems is critically maintained by the immune regulatory mechanisms of the gut-spinal cord axis (**Figure 3B**). Following SCI, a local inflammatory response is triggered, leading to the release of various inflammatory mediators, including cytokines and chemokines (Zhao et al., 2022). These mediators act not only at the injury site, but also systemically influence distant organs, such as the intestine, through the bloodstream.

#### Intestinal immune cells and spinal cord diseases

The intestine harbors diverse immune cell populations, each performing specialized functions to maintain intestinal immune homeostasis. Among these, Tregs play a central role in controlling inflammatory responses. Most intestinal Tregs are induced by FOXP3-negative traditional CD4^+^ T cells to promote a tolerance response to microbial flora and food antigens (Jacobse et al., 2021). Innate immune cells in the mucosa, such as innate lymphoid cells and mast cells, help maintain the balance between the intestinal barrier and microbiota, creating a mutually supportive ecosystem for the host and its microbial community. Antigen-presenting cells in the gut, including macrophages and DCs, are crucial components of the mucosal innate immune system. These cells maintain microbial tolerance in healthy individuals but rapidly respond to pathogens during inflammation (Flannigan et al., 2015). Additionally, intestinal immune regulatory cells located in the intestinal mucosa act as intermediaries for the influence of the gut microbiota on physiological and pathological characteristics, playing a vital role in maintaining the normal function of the human immune system and the stability of the internal environment (Li et al., 2018).

The interactions between intestinal immune cells and spinal cord diseases are complex and closely linked. SCI can alter the composition of the gut microbiota, resulting in dysbiosis, which subsequently modulates the activity and function of intestinal immune cells. These changes may influence the development and recovery of spinal cord diseases through the spinal cord–gut–immune axis (Schmidt et al., 2020; Kaul et al., 2024). In ALS, changes in the gut microbiota act as ecological factors by regulating immunity, metabolism, and neuronal signaling, thereby affecting the disease. In the SOD1 ALS animal model, depletion of the gut microbiota exacerbates disease progression, whereas in the C9orf72 model, it alleviates symptoms, indicating subtype-specific host-microbiota interactions (Kaul et al., 2024). A previous study has shown that patients with SCI experience damage to the intestinal barrier and increased bacterial translocation, along with impaired functioning of circulating monocytes, leading to a systemic inflammatory state, as evidenced by elevated levels of serum TNF-α and IL-6 (Diaz et al., 2021). Moreover, endotoxins produced by certain intestinal bacteria can enter the bloodstream through damaged intestinal barriers. This activates immune cells and generates systemic inflammatory responses, thereby adversely affecting recovery from SCI (Wilson et al., 2018; Alvarez-Mon et al., 2019).

#### Pathogenesis of cytokines and spinal cord diseases

Cytokines play a crucial role in the pathology of spinal cord diseases. Elevated levels of inflammatory factors such as TNF-α and IL-6 can affect the barrier function of intestinal epithelial cells. This leads to an increase in intestinal permeability, facilitating the entry of bacteria and toxins and further exacerbating the inflammatory response (Raue et al., 2023). Research has shown that in the early stages of EAE, the integrity of the BSCB is compromised, and this phenomenon is accompanied by infiltration of green fluorescent protein-positive myeloid cells, of which neutrophils are the predominant type. Infiltration is closely linked to changes in the BSCB permeability, and neutrophil depletion can delay the onset of EAE and reduce its severity (Aube et al., 2014).

In SCI, overexpression of inflammatory cytokines exacerbates neuroinflammatory responses, leading to further damage to the spinal cord tissue. For example, NF-κB mediates the production of chemokine (C–X–C motif) ligand 1 (CXCL1) in spinal cord astrocytes. Additionally, inflammasomes play a pivotal role in SCI and spinal cord tumors by activating caspase-1 and promoting the secretion of pro-inflammatory cytokines, including IL-1β and IL-18. These mediators drive immune-inflammatory responses and contribute to further spinal cord tissue damage (Chen et al., 2023). Moreover, patients with SCI are more likely to have intestinal metabolic disorders, such as overweight and obesity, which are directly related to low-grade chronic inflammation (da Silva Alves et al., 2013; Guerbette et al., 2023).

### Neural regulation in the gut–spinal cord axis

The complex neural network spreads throughout the human body and enables bidirectional communication between the spinal cord and the gut (**Figure 3C**). SCI is followed by varying degrees of autonomic nervous system dysregulation, which is characterized by ENS atrophy and reduced activity (White et al., 2020; Werner et al., 2023). Moreover, supplementation with probiotics and prebiotics can improve intestinal transit and prevent the pathological processes underlying ENS atrophy (Hamilton et al., 2024). Additionally, SCI leads to a maladaptive sympathetic-neuroendocrine-adrenal reflex. This reflex mediates immunosuppression due to an imbalance between glucocorticoids and catecholamines to prevent harmful infections (Pruss et al., 2017). In contrast, the parasympathetic nervous system, which originates from the midbrain, medulla oblongata, and sacral segments of the spinal cord, also regulates the contractile responses of smooth muscles during ingestion through its excitatory influences (Ali and Chen, 2023). The balance between sympathetic inhibition and parasympathetic stimulation is crucial for coordinated operation of the gastrointestinal tract. Furthermore, the autonomic nervous system interacts with the enteroendocrine system, which is controlled by the ENS (Mazzawi et al., 2020). The enteroendocrine system consists of various enteroendocrine cells (Xian et al., 2023), which can produce glucagon-like peptide-1 (GLP-1), peptide YY (PYY), cholecystokinin (CCK), substance P, and 5-hydroxytryptamine (5-HT).

#### Glucagon-like peptide-1

GLP-1 exerts substantial protective effects against cord diseases. Previous studies have shown that the administration of GLP-1 or its analogs can effectively improve neurological function in animal models of SCI (Han et al., 2020; Noguchi et al., 2024). For instance, the GLP-1 receptor agonist exenatide enhances the endoplasmic reticulum stress response and improves motor function recovery after SCI by increasing the number of macrophages with the M2 phenotype (Noguchi et al., 2024). The protective mechanisms of GLP-1 may involve inhibition of oxidative stress, inflammation, and apoptosis. In terms of oxidative stress, GLP-1 enhances the activity of antioxidant enzymes such as superoxide dismutase (SOD), reducing the production of ROS and thereby mitigating oxidative stress-induced damage to spinal cord tissue (Xue et al., 2024).

GLP-1 can also positively influence neural regeneration of spinal cord diseases. Administration of sitagliptin has been shown to significantly upregulate GLP-1 receptor protein levels following SCI, reduce neuronal apoptosis through the adenosine monophosphate (AMP)-activated protein kinase/peroxisome proliferator-activated receptor γ coactivator 1α signaling pathway, enhance axonal regeneration, and ultimately improve functional recovery (Han et al., 2020). *In vitro* experiments have demonstrated that GLP-1 promotes the differentiation of neural stem cells into neurons and increases the expression of neuronal markers. GLP-1 plays a role in spinal cord diseases through multiple signaling pathways. One of these pathways is the phosphoinositide 3-kinase-protein kinase B-Nrf2 pathway, which is a key pathway in its function. After SCI, GLP-1 can activate the phosphoinositide 3-kinase-protein kinase B pathway, which promotes the nuclear translocation of Nrf2 and activates downstream antioxidant genes, such as heme oxygenase-1, thereby enhancing cellular antioxidant capacity and reducing oxidative stress damage (Li et al., 2021). Moreover, the GLP-1 receptor agonist exenatide has been shown to stimulate the expression of microglia IL-10 and β-endorphin through Gs-cAMP/PKA/p38β/CREB and IL-10/IL-10 receptor-α/signal transducer and activator of transcription 3 signaling pathways, followed by neuroprotection and anti-damage perception (Wu et al., 2017).

#### Tryptophan and 5-hydroxytryptamine

Tryptophan, an essential amino acid in the human body, is converted into 5-hydroxytryptophan by tryptophan hydroxylase. Subsequently, 5-hydroxytryptophan is catalyzed by aromatic L-amino acid decarboxylase to form serotonin (5-HT). As a key neurotransmitter, 5-HT plays a crucial role in the regulation of various physiological functions, including mood, sleep, and appetite. In a model of severe SCI, the enzymes involved in tryptophan metabolism exhibited tissue-specific changes. Furthermore, *Limosilactobacillus reuteri* can promote the production of indole-3-carboxaldehyde by regulating tryptophan metabolism, thereby reducing neuroinflammation following SCI, improving spinal cord pathology, and facilitating motor function recovery (Boyko et al., 2021). The gut microbiota play a crucial role in tryptophan metabolism. Microorganisms in the gut can influence tryptophan metabolism through various pathways that affect the host’s physiological and pathological processes. A previous study has shown that the gut microbiota can convert tryptophan into multiple metabolites such as indole-3-acetic acid (IPA), indole, and tryptamine, which can activate aryl hydrocarbon receptors (AhRs) and thereby regulate the host immune response, intestinal barrier function, and metabolism (He et al., 2023).

Dietary fiber influences tryptophan metabolism by modulating the gut microbiota, which competes with and metabolizes tryptophan, thereby altering the production of tryptophan-derived metabolites. *In vitro* cultures and animal experiments have shown that the fiber-degrading *bacterium Bacteroides polymorphus* can inhibit indole production by providing monosaccharides to Escherichia coli, allowing more tryptophan to be utilized by Clostridium sporogenes, which produces ILA and IPA, thereby increasing their levels (Sinha et al., 2024). Additionally, in a mouse model of acute colitis induced by dextran sodium sulfate, significant changes were observed in the gut microbiota, including alterations in microbial diversity, indicator species, and the abundance of gut microbiota related to tryptophan metabolism. The tryptophan–tyrosine–kynurenic acid pathway is also dysregulated in the serum and brain, suggesting a close relationship between gut microbiota and tryptophan metabolism (Zhao et al., 2023). Therefore, therapeutic strategies targeting the regulation of tryptophan metabolism in the gut-spinal cord axis have potential value in the treatment of spinal cord diseases.

#### γ-Aminobutyric acid

In the CNS, GABA is an important neurotransmitter that acts as an inhibitor. Nevertheless, recent investigations have indicated that GABA can be generated in the gut and that its effects are mediated through regulation of the gut microbiota. The gut microbiota can influence GABA synthesis and release through multiple mechanisms. For instance, some gut bacteria such as *Lactobacillus* and *Bifidobacterium* (Yan et al., 2020) can directly produce GABA through metabolic pathways. In the gut-spinal cord axis, GABA may influence sensory input to the spinal cord by adjusting the activity of the ENS (Conn et al., 2024). Studies have shown that in the intestine, GABA can affect intestinal motor and sensory functions through its interactions with the ENS (Dicks, 2023; Liu et al., 2023). This process may entail complicated signaling among intestinal neurons and glial cells (Delvalle et al., 2018). Furthermore, GABA may modulate the conveyance of pain and other sensory signals through its impact on sensory input within the spinal cord (Slawinska et al., 2025). Thus, the gut flora may perform major regulatory functions in the gut-spinal cord axis by affecting the generation and discharge of GABA. A deeper exploration of this mechanism will help clarify the possible role of the gut flora in neurological diseases and present novel concepts for the development of relevant treatment strategies.

### Vicious cycle of gut–spinal cord interplay

A complex web of interactions is formed by gut microbiota dysbiosis, immune-mediated crosstalk, and neuroinflammation, resulting in a bidirectional feedback loop (**Figure 3D**). For example, alterations in the gut microbiota can compromise the intestinal barrier, increase its permeability, and allow toxic compounds to enter the bloodstream. These compounds trigger immune responses that amplify neuroinflammation and further disrupt gut-spinal cord communication. This creates a vicious cycle in which gut dysfunction and neurological abnormalities perpetuate chronic inflammation, barrier impairment, and autonomic nervous system dysregulation (e.g., inflammatory bowel disease, multiple sclerosis, and other diseases related to the gut and nervous system). To develop effective treatments, a thorough understanding of the complexity of these interactions, which are based on mutualism rather than one-way processes, is essential. Interrupting this vicious cycle and restoring the equilibrium of the organism in its entirety may be feasible by viewing the gut-spinal cord axis as a whole, thereby improving the patient’s quality of life (Ortega et al., 2023). Moreover, the initiating factors driving the vicious cycle at different stages of various diseases must be further investigated to enable the development of appropriate interventions.

## Evaluation of the Gut–Spinal Cord Axis

The detection methods used for studying the gut microbiota are extremely important when investigating the complex relationship between the gut and the spinal cord. A commonly used approach involves 16S rRNA and metagenomic sequencing, which provide comprehensive and in-depth insights into microbial communities. Using these methods, researchers can identify functional capabilities and clarify the metabolic pathways of microorganisms (Su et al., 2024). Moreover, these methods are highly effective in detecting low-abundance microbial species, which is crucial for gaining a comprehensive understanding of the complex interactions among gut microbes. Scientists should consider their research aims and resource limitations when selecting detection methods; 16S rRNA sequencing is appropriate for large-scale initial studies, while metagenomic sequencing is preferred for in-depth analyses of the functions and metabolism of microorganisms (Chitcharoen et al., 2025). By combining these approaches, researchers can better elucidate the roles of gut microbes in the gut–spinal cord axis. Investigating biomarkers within this axis is crucial for deepening our understanding of the underlying complex pathophysiological processes involved.

Research on biomarkers of the gut-spinal cord axis is still in its early stages, and only preliminary investigations have been conducted to date. For example, significant changes have been observed in the abundance of specific bacterial genera in individuals with SCI. The higher abundance of *Faecalibacterium*, *Megamonas*, *Prevotella*, and *Agathobacter* in patients with SCI compared with healthy controls suggests that the abundance of these bacterial subpopulations may be associated with SCI severity and the patient’s overall prognosis (Jing et al., 2023; Zhang et al., 2023c). *Faecalibacterium* exerts a protective effect on digestive health and strengthens the host immune system and intestinal barrier. Studies have shown that low levels of *Faecalibacterium* are associated with inflammation (Fritsch et al., 2021; Effendi et al., 2022). Butyrate produced by *Faecalibacterium prausnitzii* activates GPR43 to exert anti-inflammatory effects (Li et al., 2022a). In addition, a high abundance of *Megamonas* is positively associated with certain immune system diseases and may play a pro-inflammatory role; however, no specific study has explored the underlying mechanism (Balmant et al., 2023). Intestinal *Prevotella* drives chronic inflammation via TLR2 activation, induces IL-23 and IL-1 production, and promotes Th17 cell differentiation (Jiang et al., 2022). Moreover, *Agathobacter* attenuates microglial-mediated neuroinflammation through SCFAs (Lv et al., 2024).

Indicators of tryptophan metabolism have potential applications in the diagnosis of spinal cord diseases (Montgomery et al., 2022; Huang et al., 2023). Studies have shown that 5-methoxytryptophan, a tryptophan metabolite, plays a role in neuroinflammation following SCI (Hong et al., 2020; Wu et al., 2020). *In vitro* experiments have demonstrated that 5-methoxytryptophan can reduce the production of pro-inflammatory cytokines such as TNF-α, IL-1β, IL-6, and IL-18, and decrease the activation of pro-inflammatory microglia by negatively regulating the p38-mitogen-activated protein kinase signaling pathway and NLRP3/caspase-1 expression (Hong et al., 2020). Furthermore, the key enzymes and metabolites of the tryptophan metabolic pathway may serve as biomarkers for diagnosing spinal cord diseases. For instance, in the kynurenine pathway, indoleamine 2,3-dioxygenase and tryptophan-2,3-dioxygenase may undergo changes in their activities or expression levels, which could be related to the initiation and development of spinal cord diseases (Torok et al., 2020). These findings provide new perspectives and pathways for the diagnosis of these diseases.

Therefore, research on biomarkers in the gut-spinal cord axis offers substantial potential for enhancing our understanding of the fundamental mechanisms of spinal cord diseases and for devising more efficient diagnostic and treatment approaches. With advancements in research, the potential of these microbial markers to transform patient care and improve clinical outcomes is becoming increasingly clear.

## Current Status of Clinical Translation and Application

### Fecal microbiota transplantation

FMT involves the transfer of healthy, carefully screened microbial communities into a patient’s gut, restoring a balanced and resilient intestinal ecosystem and addressing both gastrointestinal disorders and systemic conditions associated with gut dysbiosis. FMT is a vital intervention. In SCI mouse models, the gut microbiota composition after FMT was shown to be similar to that in healthy mice (Xie et al., 2025). FMT has also been shown to enhance neuronal survival and axonal regeneration, improve motor function, and reduce deposition of the extracellular matrix, formation of glial scars, and inflammation. The IL-17 generated by γδ T cells is responsible for the therapeutic effects of FMT. The metabolic reprogramming in DCs induced by FMT through the interactions between DCs and Tregs also regulates γδ T cells. Moreover, the gut microbiota and its metabolites can affect the immune-inflammatory response following SCI by modulating the reactions of central and peripheral immune cells (Xi et al., 2024).

In one study, two patients with advanced ALS requiring tracheostomy and mechanical ventilation showed notable improvements in respiratory function, muscle strength, and swallowing ability after two rounds of FMT, which allowed successful weaning from the ventilator (Yan et al., 2024). Metagenomic and metabolomic analyses revealed that FMT increased the abundance of beneficial bacteria and regulated metabolic pathways, including arginine biosynthesis. This approach is a potential therapeutic option for patients with ALS and respiratory failure, and highlights gut microbiota intervention as a promising strategy for treating spinal cord diseases.

### Probiotics and prebiotics

Probiotics and prebiotics have potential applications in the treatment of diseases related to the gut-spinal cord axis (**Additional Table 1**). In animal models of SCI, the administration of probiotics can improve gut microbiota dysbiosis, reduce neuroinflammation, and promote neurological recovery. For example, feeding commercial probiotics containing lactic acid bacteria to SCI mice can trigger protective immune responses in the GALT and enhance motor function recovery (Kigerl et al., 2016). In another study, the administration of butyric acid or the probiotic VSL#3 was shown to improve intestinal and blood-brain barrier functions in ALS mice with transactive response DNA-binding protein 43 kDa mutations. This treatment reduced the expression of transactive response DNA-binding protein 43 kDa and glial fibrillary acidic protein in the spinal cord and brain, while increasing the expression of tight junction proteins. Mechanistically, the treatment restored butyric acid-producing bacteria and reduced inflammatory factors, indicating that modulation of the gut microbiota could serve as a new therapeutic strategy for ALS (Zhang et al., 2024b). Moreover, randomized controlled trials have shown that probiotics can reduce the incidence of antibiotic-associated diarrhea, and specific combinations, such as *Lactobacillus reuteri* RC-14 and *L. GR-1*, have demonstrated potential in preventing urinary tract infections (Umbrello and Esposito, 2016). Probiotics may also improve gut function and overall health by regulating the microbiota.

**Additional Table 1:** Intervention involving microbial axis in spinal cord diseases

Intervention	Target disease	Pathway	Function	Reference
FMT	SCI	NF-kB	Elevate fecal SCFAs; enhance synaptic regeneration	Jing et al., 2021
FMT	SCI	IL-17+ γδ T cells	Suppress the migration of IL-17^+^ γδ T cells; enhance hind limb motor recovery	Xi et al., 2024
FMT	SCI	TLR4/MyD88	Active inflammation and apoptosis	Rong et al., 2021
*Lacticaseibacillus paracasei Z2*.	SCI	TLR/NF-kB	Increase neuroprotective and anti-inflammatory milieu; reduce inflammatory responses	Ileninova et al., 2025
Vivomixx^TM^	MS	AhR signaling	Increase Treg cells; decrease Th1 cells	Traeger et al., 2025
*Akkermansia*	EAE	IL-17	Reduce inflammatory cells	Cox et al., 2021
*Akkermansia muciniphila*	EAE	NLRP3	Promote gut health; alleviate neuroinflammation and cognitive impairment; reduce immune responses	Li et al., 2025b
Inulin	SCI	IL-10	Alleviate ENS atrophy; prevent NBD	Hamilton et al., 2024
Ellagic acid	EAE	SCFAs	Regulate acetylation; suppress immune inflammation	Han et al., 2024
Inulin	EAE	SCFAs	Suppress immune cell infiltration; reduce IL-17/IL-6/TNF-α production	Ning et al., 2024
IPA	SCI	AKT/Nrf2	Improve axonal regeneration; promote functional recovery; enhance autophagy; inhibit pyroptosis	Chen et al., 2025b
Urolithin A	SCI	NLRP3 inflammasome	Enhance mitophagy; inhibit pyroptosis; improve functional recovery	Chen et al., 2025a
*Trichinella spiralis* ESL1 antigens	EAE	IL-10	Enhance beneficial microbiota; suppress myeloid activation; regulate immune responses	Sabljic et al., 2025

AhR: Aryl hydrocarbon receptor; EAE: experimental autoimmune encephalomyelitis; ENS: enteric nervous system; FMT: fecal microbiota transplantation; IL: interleukin; IPA: indole-3- acetic acid; MS: multiple sclerosis; MyD88: myeloid differentiation primary response protein 88; NBD: neurogenic bowel dysfunction; NF-kB: nuclear factor kappa-B; NLRP3: NOD-like receptor thermal protein domain associated protein 3; Nrf2: nuclear factor erythroid 2-related factor 2; SCFAs: short-chain fatty acids; SCI: spinal cord injury; Th: T helper; TLR: toll-like receptor; TNF-α: tumor necrosis factor-α.

Prebiotics are food components that cannot be digested by the human body and selectively stimulate the growth and activity of beneficial gut bacteria. Prebiotics have been shown to increase the number of beneficial gut bacteria and promote the production of advantageous metabolites, thereby positively affecting the gut-spinal cord axis (Tawfick et al., 2022; Guha et al., 2023). For example, the prebiotic inulin can reduce intestinal complications following SCI by preventing ENS atrophy and dysmotility after injury (Hamilton et al., 2024). Additionally, combinations of probiotics with prebiotics, known as synbiotics, may exert a synergistic effect, more effectively regulating the function of the gut-spinal cord axis and providing new strategies for the treatment of related diseases.

Probiotics influence the nervous system through two distinct pathways: (1) They produce neuroactive compounds that modulate inflammation in the CNS. These compounds may reach the brain via the circulatory system or the vagus nerve, thus regulating CNS inflammatory responses. (2) They interact with the intestinal immune system, helping to decrease the levels of pro-inflammatory cytokines such as TNF-α and IL-6, while simultaneously increasing the levels of anti-inflammatory cytokines such as transforming growth factor-β. These modulatory effects improve the immune microenvironment around the spinal cord. Furthermore, probiotics enhance intestinal barrier integrity, preventing the translocation of harmful pathogens into the bloodstream and thereby providing indirect protection to the nervous system (Umbrello and Esposito, 2016). Therefore, microbial interventions hold potential for clinical research applications. Several promising preliminary clinical trials have explored the use of microbial interventions (**Additional Table 2**), including FMT treatment, as well as the administration of probiotics and prebiotics to treat a range of CNS diseases (DuPont et al., 2023). Nevertheless, these findings have not yet been fully translated into human clinical studies (Jing et al., 2022; Xi et al., 2024), indicating that more research is needed to validate these approaches in clinical settings.

**Additional Table 2:** Clinical trials on spinal cord diseases and gut microbiota

Disease	Registration number	Intervention	Study type	Sample	Focus/outcome	Primary/secondary endpoints
SCI	NCT06870331	Probiotics + Prebiotics	Interventional	Adults with chronic SCI & bowel dysfunction	Bowel symptoms; gut microbiome composition & metabolites	Gut microbiome composition, inflammation markers, and stool frequency
	NCT03987126	Prebiotics (human milk oligosaccharides: 2'-FL + LNnT)	Randomized, double-blind, placebo-controlled (pilot)	SCI with neurogenic bowel/bladder dysfunction	Bowel motility; QoL; stool/urine microbiome changes	Bowel motility improvement, stool consistency, and QoL measures
	NCT02099890	Dietary modification (anti-inflammatory diet; high-fiber emphasis per protocol)	Interventional	Adults with SCI	Chronic inflammation & related disorders following SCI	Chronic inflammation markers, inflammatory cytokines, and biomarkers
MS	NCT04574024	High-fiber supplement (e.g., NBT-NM108)	Interventional	Adults with MS	SCFA-producing taxa; inflammation & symptoms	SCFA-producing taxa and inflammation markers
	NCT06402487	Short-chain fatty acid supplementation (propionic acid)	Randomized, double-blind, placebo-controlled	Adults with MS (add-on therapy)	Efficacy *versus* placebo; immune markers; clinical endpoints	Clinical improvement, immune markers, and MRI changes
	NCT04038541	Prebiotics *versus* Probiotics	Randomized crossover	Adults with MS	Immunologic effects; microbiome comparison	Gut microbiome comparison and immune modulation
	NCT03594487	FMT via colonoscopy (FMP30)	Phase 1b, open-label	Relapsing-remitting MS	Safety/feasibility; immune & clinical markers	Safety, immune and clinical markers
	NCT03183869	FMT	Interventional (pilot)	Relapsing MS	Inflammatory biomarkers; MRI activity	Inflammatory biomarkers, MRI activity, immune responses
	NCT03975413	FMT (donor stool)	Pilot/case series	Adults with MS	Donor/route; long-term feasibility	Donor selection, route of FMT, and clinical feasibility
	NCT05539729	Oral vancomycin	Interventional	Adults with MS	Gut-brain axis modulation via antibiotic	Gut-brain axis modulation, immune and clinical effects
ALS	NCT03766321	FMT (endoscopic; baseline + 6 mon)	Interventional	Adults with ALS	Safety; biological impact; disease-modifying potential	Safety and disease-modifying effects
	NCT07017946	Intestinal microbiome transplant capsules (MTP-101C)	Interventional (24 wk)	Adults with ALS	Safety/tolerability; NfL biomarker; and clinical outcomes	Safety/tolerability, biomarkers, and clinical outcomes
	NCT06051123	Probiotics versus placebo	Randomized controlled	Adults with ALS	Lipidomic/metabolic changes; clinical outcomes	Lipidomic/metabolic changes, and clinical outcomes
	NCT03324399	Investigational probiotic (with microbiome profiling)	Exploratory (small sample)	Adults with ALS	Amino acids pre/post-prandial; longitudinal microbiome	Microbiome profiling, pre/post- prandial amino acid levels

ALS: Amyotrophic lateral sclerosis; FMT: fecal microbiota transplantation; MRI: magnetic resonance imaging; MS: multiple sclerosis; QoL: quality of life; SCFA: short-chain fatty acid; SCI: spinal cord injury.

### Spinal cord injury-microbiota mediator: short-chain fatty acids

SCFAs can restore the BSCS, enhance the survival rate of neurons, facilitate axon formation, reduce astrocyte proliferation, and inhibit microglial activation, thereby promoting the recovery of motor function in SCI mice. SCFAs, such as acetate (C2), propionate (C3), and butyrate (C4), are saturated fatty acids containing fewer than six carbon atoms, and are mainly produced by the fermentation of dietary fiber by bacteria in the intestine (Canani et al., 2011). In a study evaluating a large cohort of Italian patients with SCI in the acute phase and age- and sex-matched healthy controls (Bazzocchi et al., 2021), the patients with SCI exhibited increased levels of potentially pathogenic, pro-inflammatory, and mucus-degrading bacteria, along with a reduction in SCFA-producing bacteria. SCFAs and NF-κB signaling may even mediate limb function promotion, neuronal axon regeneration, and recovery of intestinal barrier integrity and gastrointestinal motility in SCI mice (Jing et al., 2021), which underscore the potential of microbial interventions as a key therapeutic tool for SCI. In conclusion, gut health can alleviate the negative effects of SCI on systemic health through immunomodulation, glial scar formation, and axonal regeneration. These findings provide novel and promising therapeutic targets for SCI.

### Nanomaterials and extracellular vesicles

The major advances in gut health and tissue engineering have greatly improved the delivery methods for functional microbe- and cell-based therapies. One subset of these technological advances involves the development of new probiotic formulations that improve gut colonization. For example, artificial microparticles of specific probiotics have been engineered to enhance gut colonization. These particles are designed to survive the harsh gastric environment and effectively release their contents into the intestine, thereby maximizing their beneficial effects on digestive health. Polysaccharide gel delivery systems represent another novel method. These systems are carefully designed to guarantee precise targeted release of probiotics in the intestine. Using this approach, probiotics can achieve their optimal concentration at the site of action, thus maximizing their therapeutic potential. In addition, these systems can be customized to release probiotics over a long period of time, thereby offering continuous benefits.

Nanotechnology has substantially enhanced the bioavailability of both probiotics and prebiotics. By reducing these particles to the nanoscale, their surface area can be significantly increased, thereby improving their absorption and efficacy. This approach effectively overcomes many traditional challenges in delivering probiotics and prebiotics (Dangi et al., 2023). Dual-coating encapsulation technology is another approach that has yielded substantial progress in shielding probiotics from the severe acidic conditions of the stomach. This technology coats probiotic cells with two protective layers, which markedly enhances their survival in gastric acid. In some cases, it has increased probiotic survival rates by over 100-fold, ensuring that a substantially greater proportion of cells reach the intestines alive to exert beneficial effects. These advances in gut-targeted delivery and tissue regeneration highlight the transformative potential of these technologies for improving patient outcomes.

**Figure 4:**
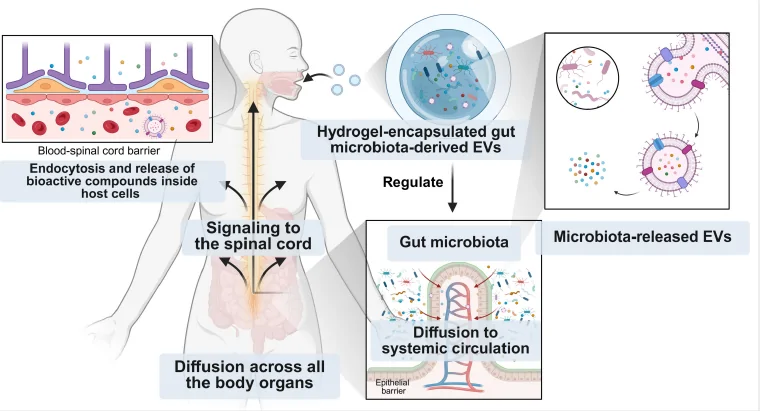
Microbiota-derived extracellular vesicles (EVs) as an oral delivery system for host-microbiome interaction. The proposed pathway mechanism involves microbiota-released EVs functioning as an oral delivery system with nanomaterials, mediating host–microbiome communication and transporting bioactive metabolites.

Crucially, considering the difficulty in penetrating the BSCS, the oral administration of gut microbiota-derived EVs combined with nanomaterials has shown potential for spinal cord regeneration. This method harnesses the natural biological activity of EVs and the excellent delivery characteristics of nanomaterials to enhance nerve regeneration and functional recovery after SCI (**Figure 4**). Gut microbiota EVs have demonstrated significant effects on the regulation of inflammatory responses and the promotion of tissue regeneration. EVs secreted by the gut microbiota have been shown to improve recovery after SCI by modulating the immune microenvironment and promoting neuronal regeneration (Liu et al., 2020; Rong et al., 2024). Moreover, EVs can directly access the CNS through the blood–brain barrier, offering unique benefits in the treatment of neurological injuries (Mottawea et al., 2025).

Additionally, the use of EV delivery systems can provide stability and targeting properties to EVs. Nanomaterials can shield EVs from rapid clearance and enhance their accumulation and controlled release into target tissues through specific modifications (Cui et al., 2025). Finally, the delivery of exosomes via oral intake is feasible for easy application. Compared with invasive injections, oral administration is more convenient and patient-friendly, resulting in higher compliance. The protection provided by nanomaterials allows exosomes to maintain their activity in the gastrointestinal tract for successful absorption and targeted delivery (Cui et al., 2024a). Overall, the oral administration of EVs derived from gut microbiota, in conjunction with nanomaterials, holds promising prospects for application in the treatment of spinal cord regeneration and can effectively enhance neural regeneration.

### Integrated treatment of the gut–spinal cord axis

**Figure 5:**
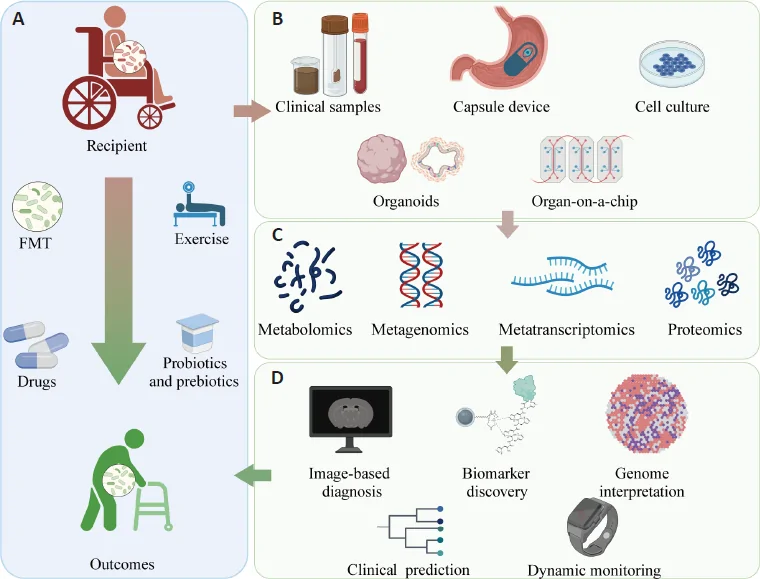
Schematic illustration of prospect and future application. (A) Various interventions contribute to promoting spinal cord injury recovery through the gut–spinal cord interaction. (B) Current and potential future personalized sample studies. (C) Using multi-omics tools to study the relationship between gut and spinal cord and build a personalized biological database for data analysis. (D) Utilizing artificial intelligence for future data analysis, including image-based diagnosis biomarker discovery, genome interpretation, clinical prediction and dynamic monitoring. Created with BioRender.com. FMT: Fecal microbiota transplantation.

Given the complexity of the gut–spinal cord axis, a comprehensive treatment approach may be effective in improving related diseases (Ju et al., 2025; **Figure 5**). FMT, as an emerging therapy, has shown potential in SCI treatment (Xie et al., 2025). Studies have shown that FMT can modulate the gut microbiota, improving inflammatory responses and neurological recovery after SCI, possibly through its effects on the functions of immune cell such as γδ T cells (Jing et al., 2022; Xi et al., 2024). Moreover, the combination of probiotics and prebiotics with regenerative interventions for SCI may provide synergistic benefits. For instance, the combination of therapeutic agents for regulating the gut microbiota with stem cell transplantation or neuroregenerative drugs could more effectively restore the gut–spinal cord axis function, thereby enhancing neurological recovery (Tan et al., 2023). Simultaneously, rehabilitation therapy, including physical therapy and exercise training, can also be considered to further enhance the patient’s quality of life and provide a more comprehensive approach to treating diseases related to the gut–spinal cord axis (Li et al., 2025c). Future clinical trials should aim to address key questions, including microbial strain specificity, donor selection, optimal timing, and integration of microbial therapies. Moreover, strategies that combine microbial interventions with non-microbial therapies warrant further investigation.

## Challenges and Future Prospects of Research on the Gut–Spinal Cord Axis

### Application of emerging technologies in the study of the gut–spinal cord axis

Emerging technologies can present novel methods for studying the gut–spinal cord axis (**Figure 5**). The evolution of metagenomics is expected to provide a more comprehensive view of the complex relationships between the gut microbiota and the CNS, which may yield novel insights into the effects of these microbial groups on the health and function of the spinal cord (Du et al., 2021).

Single-cell sequencing technology can analyze gene expression and function at the single-cell level in the gut microbiota and spinal cord cells, providing a more detailed understanding of their interactions (McCallum and Tropini, 2024). Using this technology, researchers can identify specific microbial species and their relevant genes that actively participate in communication with the spinal cord cells. This in-depth knowledge can facilitate the targeted use of probiotics and prebiotics to regulate the gut microbiota, which may improve spinal cord health and function.

Advances in sample research can also drive the implementation of personalized medicine. At present, human and murine fecal extracts are the most common sources of samples for gut microbiota research. However, these samples merely represent the microbial makeup of the rectum and sigmoid colon (Cizkova et al., 2021), making them prone to contamination by environmental microorganisms and restricting the portrayal of regional intestinal physiology. To address this issue, scientists have created a capsule-based device that enables the collection of luminal substances from different parts of the gastrointestinal tract for multi-omics analysis (Shalon et al., 2023). The capsule, which is furnished with detectors and sample-gathering parts, can be ingested by the subjects and subsequently travels through the digestive tract, gathering specimens at pre-set points. Using such samples, researchers can gain a more comprehensive understanding of microbial makeup and function; moreover, by analyzing these samples, researchers can determine how they interact with the host in various intestinal regions by using multi-omics techniques such as metagenomics, metabolomics, and proteomics. In addition to overcoming the limitations of rectal and sigmoid colon samples, this approach can also offer a significant understanding of the differences in gut microbiota across regions and their possible influences on health and disease.

With the growing number of sample sources, the extraction and handling of sample data using machine learning models will become a critical step in exploring the gut-spinal cord axis. This revolutionary finding is expected to establish a new standard for evaluating the condition of the human gut, promote the development of personalized microbial detection techniques, and provide a powerful tool for clinical disease prevention and public health management (Wu et al., 2024a).

Organoid development may become an important source of biological samples in the future. Intestinal organoids can facilitate disease modeling and drug screening and also hold potential applications in regenerative medicine. Research on spinal cord organoids is currently advancing. Using human induced pluripotent stem cells to generate spinal cord organoids, researchers can better simulate the three-dimensional organization and physiological functions of the spinal cord, providing a fundamental platform for studying spinal cord diseases and developing new therapeutics (Wang et al., 2024b). Moreover, the gut environment can be modeled more adequately by incorporating probiotics and prebiotics into the organoid cultures and testing them to gain insights into their influence on gut health and, potentially, spinal cord function through the gut-spinal cord axis. Additionally, one study engineered a 3D axis-on-a-chip platform using microfluidic technology to link visceral sensory ganglion organoids with human colonic organoids, thereby establishing functional neural connections (Ahn et al., 2024). This system can substantially enhance mechanistic studies of the gut–nervous system axis. The proposed approach holds promise for personalized medicine, allowing patient-specific probiotic or prebiotic interventions based on personalized gut microbiome features.

Over the past few years, remarkable advances have been made in the use of nanomaterials for oral drug delivery. Nanomaterials are widely used in the pharmaceutical and dietary industries owing to their good biocompatibility, excellent protective properties, drug-releasing capabilities, and targeting abilities (Li et al., 2024c). These substances are efficient in safeguarding drugs against degradation in the gastrointestinal tract, boosting their stability and intestinal absorption and thereby enhancing their bioavailability. Furthermore, nanomaterials can promote the colonization of probiotics by modulating gut microbiota. Further research and clinical verification are required to guarantee the safety and effectiveness of nanomaterials in real-world applications (Dass et al., 2025).

Future SCI research should take advantage of advancements in personalized rehabilitation strategies, omics technologies, nanomaterials, and applications of artificial intelligence to optimize treatment methods and improve patient outcomes (Shalon et al., 2023; Wang et al., 2024a). This field is poised to unlock new frontiers in SCI management and recovery by harnessing the potential of gut microbiota-targeted interventions and integrating multi-omics data (Chang et al., 2023).

### Existing challenges and limitations in studies of the gut–spinal cord axis

Despite some progress in studies of the gut–spinal cord axis, several controversies remain. The causal relationship between gut microbiota and spinal cord diseases has not been fully clarified. Although numerous studies have demonstrated an association, they have not clarified whether gut microbiota dysregulation drives spinal cord diseases, whether spinal cord diseases induce alterations in the gut microbiota, or whether other complex interactions are involved. Thus, more research is required to elucidate these mechanisms (Guha et al., 2023; Li et al., 2025c).

Another major limitation is the lack of longitudinal human studies and reliable biomarkers. Most current treatment methods are still in the experimental stage, and their efficacy and safety require further validation. Although FMT has demonstrated promising results in animal studies, its clinical application still faces challenges related to donor selection, standardization of transplantation procedures, and long-term safety (Xi et al., 2024). Moreover, an understanding of the regulatory mechanisms of the gut–spinal cord axis is lacking, especially the specific regulatory mechanisms under different disease backgrounds, which limits the development of targeted therapeutic strategies.

Oral administration is often limited by poor colonization by probiotics and low bioavailability of drugs, prebiotics, or postbiotics due to multiple influencing factors. Oral probiotics and prebiotics must survive in the acidic environment of the stomach and resist degradation by digestive enzymes, which diminishes their activity and effectiveness (Zhang et al., 2023a). Furthermore, the liver’s purification process also affects the bioavailability of oral medications. Before entering systemic circulation, oral drugs usually undergo first-pass metabolism in the liver, which may greatly decrease the effective concentration of the drug (New, 2020). Finally, the effects of certain drugs and probiotics on the CNS are restricted by the blood-brain barrier. The blood-brain barrier selectively filters substances, preventing most large molecules and microbes from entering the CNS and thereby limiting their use in treating neurological disorders (Lee et al., 2021). In summary, multiple physiological barriers restrict the colonization and bioavailability of probiotics and prebiotics when administered orally. Some obstacles can be partially overcome and their treatment effects can be enhanced by improving drug delivery systems by using approaches such as nanotechnology and colloid encapsulation (Rose et al., 2021; Zhang et al., 2023a).

### Directions for future research in the gut–spinal cord axis

The gut–spinal cord axis involves complex regulatory mechanisms that warrant further investigation. Future research should focus on clarifying the bidirectional interactions between the gut microbiota and the spinal cord, including signaling pathways and immune regulatory mechanisms, to elucidate how this axis functions in both health and disease (Kigerl et al., 2020).

Additionally, research on biomarkers associated with the gut–spinal cord axis should be enhanced. More precise and reliable biomarkers are needed for the early diagnosis of diseases, condition monitoring, and prognosis evaluation. Research on therapeutic approaches should concentrate on developing more effective strategies and optimizing existing treatments. For example, improving the efficiency of microbial interventions is a critical goal. Furthermore, the interactions between the gut-spinal cord axis and other physiological systems should receive attention. This broader perspective can clarify the impact of this axis on overall health and support a comprehensive strategy for managing related diseases.

## Conclusions

The gut–spinal cord axis represents a transformative framework for understanding the interplay between gut health and spinal cord pathophysiology. This bidirectional network integrates microbial, immune, neural, and metabolic pathways to regulate gastrointestinal function, spinal cord homeostasis, and systemic inflammation. The gut-spinal cord axis is significant for future clinical applications and rehabilitation. In spinal cord diseases, gut dysbiosis disrupts SCFA production and immune equilibrium, exacerbating intestinal barrier dysfunction and neuroinflammation, while autonomic neuropathy further amplifies gut dysregulation. Despite cpnsiderable advances in preclinical models, such as the neuroprotective roles of SCFAs, EV-based nanodelivery systems, and microbiota modulation, translational challenges persist, including mechanistic uncertainties and therapeutic scalability. Future research should prioritize longitudinal studies to clarify causality, refine biomarkers for early diagnosis, and develop personalized interventions that account for demographic, dietary, and genetic variabilities. Innovations in multi-omics integration, organoid models, nanomaterials, and artificial intelligence-driven analytics hold promise for decoding the complexity of the gut-spinal cord axis and advancing precision medicine for SCI care. By bridging basic science and clinical practice, the gut-spinal cord axis paradigm offers a shift in rehabilitation strategies, emphasizing holistic gut-spinal cord health as a cornerstone for optimizing patient recovery and quality of life.

## Additional files:

***Additional Table 1:*** *Intervention involving microbial axis in spinal cord diseases.*

***Additional Table 2:*** *Clinical trials on spinal cord diseases and gut microbiota.*

## Data Availability

*All relevant data are within the paper and its Additional files.*
